# Mutations of *Francisella novicida* that Alter the Mechanism of Its Phagocytosis by Murine Macrophages

**DOI:** 10.1371/journal.pone.0011857

**Published:** 2010-07-29

**Authors:** Xin-He Lai, Renee L. Shirley, Lidia Crosa, Duangjit Kanistanon, Rebecca Tempel, Robert K. Ernst, Larry A. Gallagher, Colin Manoil, Fred Heffron

**Affiliations:** 1 Department of Molecular Microbiology and Immunology, Oregon Health & Science University, Portland, Oregon, United States of America; 2 Virogenomics, Inc., Tigard, Oregon, United States of America; 3 Department of Microbial Pathogenesis, University of Maryland Dental School, Baltimore, Maryland, United States of America; 4 Department of Genome Sciences, University of Washington, Seattle, Washington, United States of America; Institut Pasteur, France

## Abstract

Infection with the bacterial pathogen *Francisella tularensis tularensis* (*F. tularensis*) causes tularemia, a serious and debilitating disease. *Francisella tularensis novicida* strain U112 (abbreviated *F. novicida*), which is closely related to *F. tularensis*, is pathogenic for mice but not for man, making it an ideal model system for tularemia. Intracellular pathogens like *Francisella* inhibit the innate immune response, thereby avoiding immune recognition and death of the infected cell. Because activation of inflammatory pathways may lead to cell death, we reasoned that we could identify bacterial genes involved in inhibiting inflammation by isolating mutants that killed infected cells faster than the wild-type parent. We screened a comprehensive transposon library of *F. novicida* for mutant strains that increased the rate of cell death following infection in J774 macrophage-like cells, as compared to wild-type *F. novicida*. Mutations in 28 genes were identified as being hypercytotoxic to both J774 and primary macrophages of which 12 were less virulent in a mouse infection model. Surprisingly, we found that *F. novicida* with mutations in four genes (*lpcC, manB*, *manC* and *kdtA*) were taken up by and killed macrophages at a much higher rate than the parent strain, even upon treatment with cytochalasin D (cytD), a classic inhibitor of macrophage phagocytosis. At least 10-fold more mutant bacteria were internalized by macrophages as compared to the parent strain if the bacteria were first fixed with formaldehyde, suggesting a surface structure is required for the high phagocytosis rate. However, bacteria were required to be viable for macrophage toxicity. The four mutant strains do not make a complete LPS but instead have an exposed lipid A. Interestingly, other mutations that result in an exposed LPS core were not taken up at increased frequency nor did they kill host cells more than the parent. These results suggest an alternative, more efficient macrophage uptake mechanism for *Francisella* that requires exposure of a specific bacterial surface structure(s) but results in increased cell death following internalization of live bacteria.

## Introduction

*Francisella tularensis* is a Gram-negative facultative intracellular pathogen and the causative agent of tularemia. Four subspecies of *F. tularensis* are recognized according to Bergey's Manual of Systematic Bacteriology [1]: *F. tularensis* subsp. *tularensis* (type A), *F. tularensis* subsp. *holarctica* (type B), *F. tularensis* subsp. *mediasiatica*, and *F. tularensis* subsp. *novicida. F. novicida* strain U112 is pathogenic for mice, but not for man and the genes are on average >95% identical to *F. tularensis* strain SchuS4, making it an acceptable model system. Another advantage of working with *F. novicida* U112 is the availability of a comprehensive transposon library containing two insertion alleles for the majority of non-essential genes [2].

Lipopolysaccharide (LPS) is the major structural component of the outer membranes of all Gram-negative bacteria and recognition of this unique structure by the host is a key factor in activating a robust immune response. LPS is a tripartite macromolecule comprised of lipid A, the core, and an O-antigen and is assembled sequentially beginning with lipid A. Lipid A anchors the LPS in the outer membrane and is a disaccharide of glucosamine that is both acylated and phosphorylated [3]. The core and O-antigen carbohydrate domains are linked to the lipid A moiety through the eight-carbon sugar 3-deoxy-D-manno-octulosonic acid (Kdo) [3]. *Francisella* (and other bacteria) begin with synthesis of di-phosphorylated tetra-acylated lipid IVA in the inner leaflet of the inner membrane. In *Francisella*, this structure is initially penta-acylated and transferred to the outer leaflet of the inner membrane or, alternatively, the core sugars can also be added before the transfer [4]. Two Kdo (a disaccharide) are transferred to lipid A by KdtA while the molecule is still on the inner leaflet of the inner membrane. Once transferred to the periplasm, the nascent chain is dephosphorylated at the 4′ position by *lpxF* and one of the two Kdo saccharides is removed by Kdo hydrolase followed by the addition of mannose di-saccharide to form the core [3], [5]. The structure is transferred to the outer membrane by the Lpt ABCFG transport system. In the outer membrane, one of the acyl groups is removed via the 3′ O-acylase, leaving a tetra-acylated form. Two of the genes described in this report, *wbtA* and *wzx*, are both involved in the synthesis of the O-antigen [3]. WbtA functions as a dehydratase and is likely required for the synthesis of the first sugar attached to the core and thus all subsequent sugars as well [6], [7]. Based on homology with *E*. *coli*, the O-antigen flippase, encoded by *wzx*, translocates individual carbohydrate residues from the cytosol to the periplasm where Wzy polymerizes them into the O-antigen, which is then transferred to the core [8]. Strains that lack *wbtA* or *wzx* synthesize a LPS that lacks an O-antigen [8], [9].

The lipid A moiety without the attached Kdo unit represents the minimal LPS substructure required for bacterial viability in *Francisella* subspecies [10]. Compared to the *E. coli* or *Salmonella* LPS, the *Francisella* LPS is a much less potent endotoxin and does not stimulate inflammatory pathways via TLR4 signaling. The lack of proinflammatory response is related to hypoacylation and the addition of very long chain fatty acids attached to the diglucosamine backbone [11]. The absence of a phosphate at the 4′ position is also important as compared with LPS from enteric organisms. In *E. coli*, removal of the 4′ phosphate reduces lipid A toxicity [12].

**Figure 1:**
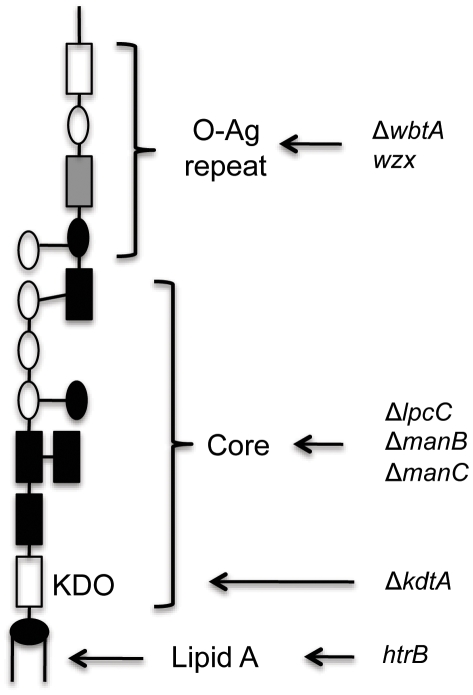
*Francisella* LPS structure of lipid A and core. Schematic drawing of a portion of the LPS including lipid A, the core and one O-antigen repeat (Modified from [4]). Indicated in the drawing are the regions of the LPS that are affected by the mutant alleles described in this study. Both *wzx* and *htrB* contain transposon insertions while the remaining mutations are gene deletions.

While the LPS biosynthesis genes have been only partially characterized in *Francisella*, the orthologous genes in *E. coli* and *Salmonella* are well characterized and likely have the same function. The addition of mannose to the core of the LPS requires the action of at least three genes, *lpcC*, *manB* and *manC* (**Figure 1**). Group 1 glycosyl transferase (*lpcC*) adds a mannose unit to the inner Kdo moiety of the LPS precursor in *E. coli*. Among the genes required for the mannose pathway in *E. coli*, *manB* (*pmm* or *rfbk*) encodes phosphomannomutase, which converts mannose-6-phosphate to mannose-1-phosphate while *manC* encodes a mannose-1-phosphate guanylyltransferase, which catalyzes mannose-1-phosphate to GDP-D-mannose (the substrate for the mannose glycosyltransferase, LpcC). Without *lpcC*, *manB*, and *manC*, mannose would not be added to the LPS structure thereby preventing the addition of the O side chains and leaving Kdo as the outer most polysaccharide [13,this report].

*Francisella* is readily phagocytosed by macrophages by an actin-dependent mechanism [14]–[16]. Following phagocytosis, *F. tularensis* strains replicate intracellularly and eventually kill both cultured macrophage cell lines and primary macrophages [15], [17]–[19]. In the current study, mutations in 28 genes identified from the comprehensive *F. novicida* transposon library were found to increase cell toxicity upon infection of J774 macrophage-like cells. To determine whether the mutant strains must be internalized by the macrophages to promote cell death, we inhibited actin polymerization by adding cytochalasin D (cytD), a classic inhibitor of macrophage phagocytosis, prior to cell infection. Surprisingly, three of the mutant strains (*lpcC, manB, manC*) were cytotoxic to macrophages treated with cytD. In-frame deletions that removed more than 90% of the coding sequence and were designed to not interfere with expression of downstream genes in the same operon were constructed in these and other genes involved in LPS biosynthesis. Mutations in only four genes were cytotoxic to macrophages treated with cytD: *kdtA* (Kdo transferase) and the three identified during the screen for hypercytotoxic mutants (*lpcC*, *manB* and *manC*). All four of these genes are required for LPS core biosynthesis.

## Results

### One class of *F. novicida* mutants kills J774 cells faster than the parental strain U112

Like other intracellular pathogens, intracellular replication of *Francisella* eventually kills the host cell. Bacteria are released following host cell death, allowing them to infect other cells. *F. novicida* type strain U112 has been known to cause damage to J774 monolayers at 18 hours post infection [20]. By directly visualizing morphological changes of infected J774 macrophages, we screened an arrayed *F. novicida* library of more than 3000 mutants containing two allelic mutations of most non-essential genes [2]. Twenty-eight transposon mutants killed J774 cells within 12 hours of infection (**Table 1**) while the parental strain U112 required 24 hours to result in substantial cell death. The degree of cytotoxicity among the 28 mutants was determined by measuring the release of lactate dehydrogenase (LDH), a cytoplasmic protein commonly used to identify cell lysis. Twelve hours after infecting J774 macrophages with approximately the same number of bacteria, cell culture supernatants were removed and assayed for LDH. The 28 hypercytotoxic mutants killed J774 macrophage-like cells more quickly (within 12 hours post infection) in comparison to the wild-type strain U112 (Column 3 in **Table 1**). *In vitro* growth did not differ among the hypercytotoxic mutants (Column 5 in **Table 1**), nor did they display any other obvious phenotype. Among the genes identified, 12 were *pil* genes involved in pilus production and protein secretion and four were involved in LPS synthesis (*htrB*, *lpcC*, *manB* and *manC*).

**Table 1:** List of novel hypercytotoxic mutants in the 2-allele library.

FTN#	Gene	LDH%^a^	Mice survival^b^	IVGI^c^
U112	Wild-type	7.7	1/3	1
**0% survival group (4)**				
FTN0946	*pilF*	73.4	0/3	1
FTN1138	*pilP*	68.7	0/3	0.9
FTN1139	*pilO*	75.2	0/3	0.98
FTN1140	*pilN*	80.3	0/3	0.94
**Partial survival group (12)**				
FTN0070	*pilE*	95.6	1/3	0.86
FTN0415	*pilA*	89.9	1/3	0.94
FTN0429	unknown	64.9	1/3	1.01
FTN0642	*cydD*	80.5	1/3	0.9
FTN0672	*secA*	100	2/3	0.84
FTN0756	*fopA*	78.3	2/3	0.89
FTN1000	*pilD*	14.3	1/3	1.07
FTN1115	*pilB*	71.2	1/3	0.9
FTN1116	*pilC*	71.1	2/3	0.9
FTN1137	*pilQ*	78.2	2/3	0.95
FTN1141	*pilM*	73.9	2/3	1
FTN1681	*fur*	19.4	1/3	0.95
**100% survival group (12)**				
FTN0071	*htrB*	100	3/3	0.87
FTN0355	*tolB*	58.9	3/3	0.78
FTN0408	unknown	16.6	3/3	0.89
FTN0528	*lpxH*	100	3/3	0.97
FTN0558	*ostA1*	100	3/3	1
FTN0664	*fimT*	66.6	3/3	1.32
FTN0757	unknown	25	3/3	0.87
FTN1253	*lpcC*	76.2	3/3	0.93
FTN1254	unknown	35.6	3/3	0.76
FTN1417	*manB*	87.7	3/3	0.9
FTN1418	*manC*	71.9	3/3	0.86
FTN1661	*nusA*	26.5	3/3	0.92

a J774 cells were infected with MOI of 200. 50 µl of supernatant was taken at 12 hours p.i. and assayed for LDH; experiment was repeated once with similar results.

b mice were infected with 60 CFU and survival was monitored for 28 days.

c *in vitro* growth index (IVGI) was done at 18 hours; experiment was repeated once with similar results.

### Twelve *F. novicida* hypercytotoxic mutants are attenuated in mice

The virulence of *Francisella* is connected to its ability to evade the immune response of a host. By increasing the rate of macrophage cell death, an infection with the hypercytotoxic mutants may induce a proinflammatory response and as a result, diminish the virulence of the strain. In order to determine whether any of the hypercytotoxic mutants are attenuated, BALB/c mice were infected with each of the 28 transposon mutant strains. Mice were inoculated intraperitoneally (i.p.) with 1 LD_50_ (60 CFUs) and monitored for survival. After 28 days, the survival rate for 12 of the 28 hypercytotoxic mutants was 100% (Column 4 in **Table 1**), suggesting that they are attenuated for virulence. The remaining 16 had lower survival rates that were more similar to the survival rate of mice infected with U112. The transposon strain with a mutation in *pilP* (FTN1138) appeared hypervirulent, as it killed all three mice within two days. Typically, mice infected with the parental strain began dying three to four days after infection.

The LD_50_ of the 12 mutant strains showing attenuation in the initial screen was determined by infecting mice with increasing doses of each mutant strain. All 12 of the mutants had a LD_50_ that was at least one order of magnitude higher than the parental strain U112 (**Table 2**). Mutants identified to be hypercytotoxic *in vitro* were more likely to be avirulent (12 of 28 strains, 43%) as compared to transposon insertion mutations screened at random for virulence in which 4–6% of insertion mutations were avirulent [21].

**Table 2:** Estimated LD_50_ of the attenuated mutants^a^.

FTN#	Gene	Estimated LD_50_	Corresponding FTT#
U112 (wt)		≤6×10^1^	NA
FTN0071	*htrB*	6.25×10^7^	FTT0231c
FTN0355	*tolB*	6.5×10^3^	FTT0840
FTN0408	unknown	6.5×10^3^	(FTT0882)
FTN0528	*lpxH*	6.0×10^2^	FTT0436c
FTN0558	*ostA1*	6.25×10^6^	FTT0467
FTN0664	*fimT*	6.5×10^2^	FTT1314c
FTN0757	unknown	7.5×10^4^	(FTT0584)
FTN1253	*lpcC*	6.75×10^3^	FTT1235c
FTN1254	unknown	6.25×10^2^	FTT1236
FTN1417	*manB*	6.75×10^3^	FTT1447c
FTN1418	*manC*	6.75×10^2^	FTT1448c
FTN1661	*nusA*	6.25×10^6^	FTT0049

a 3–5 mice were used for each dilution and survival was monitored for 28 days.

### Role of cell contact in cytotoxicity

*Francisella* is less inflammatory than many pathogens in part because its LPS is not recognized by TLR4. An alternative, although less likely scenario, is that *Francisella* secretes proteins that function to inhibit cell death. For example, as hypothesized in Hager et al. [22], secreted proteases could remove cell surface proteins (e.g. Fas and TNFaR) that are involved in signaling the cell to undergo apoptosis. Therefore, to determine if cell contact between the bacteria and macrophages is necessary for cell death, we divided a transwell (Nunc 137044) with a membrane containing 0.22 µm pores (too small for bacterial passage); on one side we grew macrophages, and on the other side we inoculated bacteria. We did not observe any increase in cell death when macrophages were separated from the wild-type U112 or mutant bacteria, indicating that contact with bacteria was necessary for cell death.

**Figure 2:**
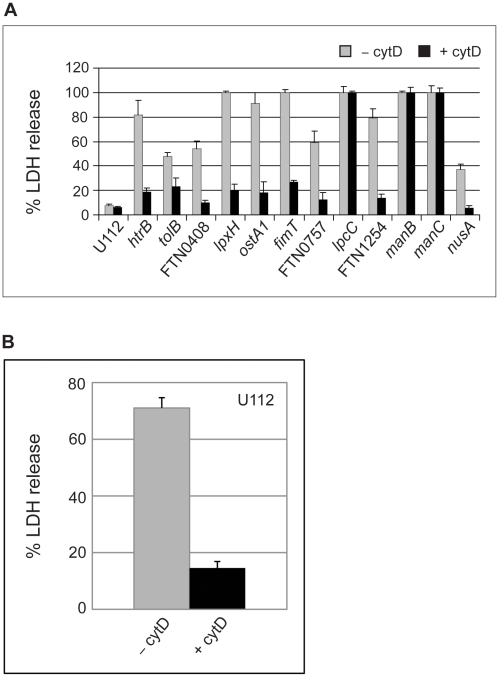
The presence of cytochalasin D (2 µM) during infection decreased LDH release in all but three of the J774 macrophage-like cells infected with *F. novicida* transposon mutant strains. (**A**) J774 macrophages were infected with each of the 12 hypercytotoxic transposon mutants or wild-type U112 either in the presence or absence of cytochalasin D (cytD). The levels of LDH in the extracellular medium were determined 12 hours post-infection (p.i.). The levels of LDH release from the mutant- or U112-infected J774 cells were normalized to the level of LDH release from uninfected macrophages lysed with detergent. Parent strain U112 does not promote cell death at 12 hours p.i. (**B**) The level of LDH release from J774 macrophages infected with U112 at a MOI of 100 was determined 24 hours p.i. LDH release was determined from macrophages infected with U112 both in the presence and absence of cytD. In (**A**) and (**B**), each column is an average of three individual infections (±s.d.). The experiment was repeated twice and yielded similar results.

Next, in order to determine whether internalization of the mutant *Francisella* strain is required for macrophage cell death, we inhibited actin polymerization by adding cytochalasin D (cytD) before and during infection with the 28 transposon mutants and U112 in J774 macrophages. CytD has been previously shown to be an inhibitor of phagocytosis of *Francisella* [14]–[16], [23]. Three of the 28 transposon mutant strains (*lpcC, manB and manC*) were highly cytotoxic when infection took place in the presence of cytD (**Figure 2A**). For the remaining mutants, the cell toxicity was greatly reduced following treatment with cytD (**Figure 2A**). In order to observe differences in U112-infected macrophages, LDH release was quantified after 24 hours. As shown in **Figure 2B**, cytD significantly reduces the cell toxicity of the parent strain 24 hours p.i.

### Internalization of three mutant strains occurs independently of actin polymerization

**Figure 3:**
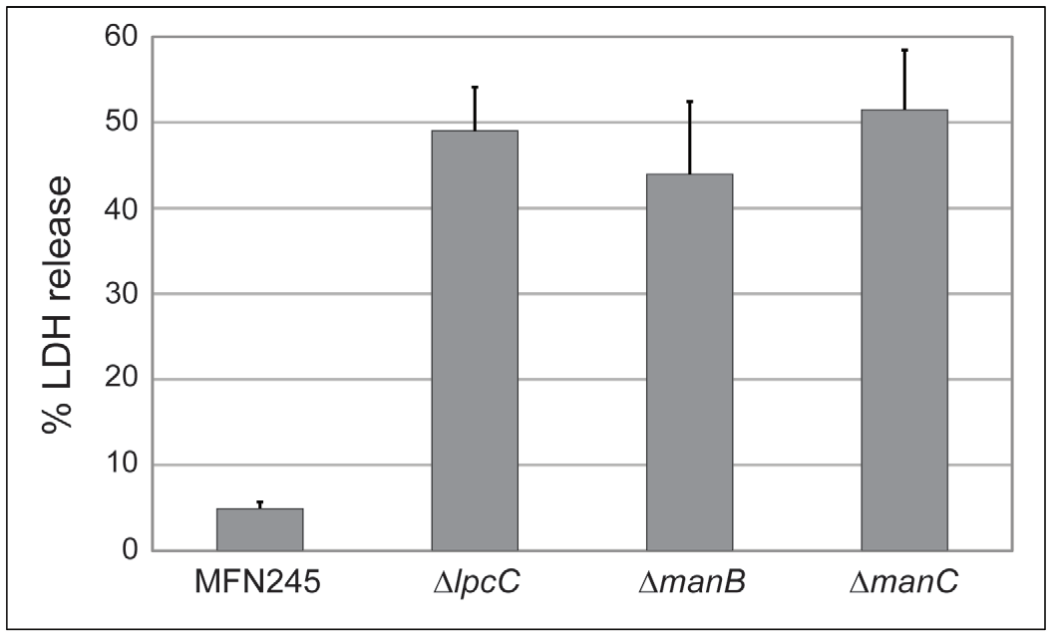
Strains containing deletion mutations in *lpcC*, *manB*, and *manC* induce early cytotoxicity in primary macrophages. Bone marrow-derived macrophages (BMDM) derived from BALB/c mice were infected with the deletion mutants or parental strain MFN245 at a MOI of 100. The level of LDH release from infected macrophages was determined 10 hours p.i. as described in Figure 2. Each column is an average of three individual infections (±s.d.). Repetition of this experiment yielded similar results.

The three transposon mutant strains that remained hypercytotoxic in the presence of cytD contained transposon insertions in genes necessary for biosynthesis of LPS core (*lpcC, manB*, and *manC*). Transposon insertions can have polar effects on downstream genes, and *manB* and *manC* are within an operon; therefore, we constructed deletions of *lpcC*, *manB*, and *manC* in the U112-based restriction-deficient strain MFN245 (see Materials & Methods). In this background, we confirmed that individual deletions of these genes still result in a hypercytotoxic phenotype that is independent of actin polymerization. LDH release from J774 macrophages infected with the deletions of FTN1253 (Δ*lpcC*), FTN1417 (Δ*manB*), and FTN1418 (Δ*manC*) with a multiplicity of infection (MOI) of 100 or 1000 was significantly (p<0.01) higher than macrophages infected with the same MOI of the parent strain and similar to the LDH release observed after infection with the transposon mutants (see below). LDH release from macrophages infected with MFN245 was comparable to LDH release from macrophages infected with wild-type U112. Furthermore, the presence of cytD did not affect LDH release in J774 macrophages infected with Δ*lpcC*, Δ*manB*, and Δ*manC* (data not shown). To confirm that the hypercytotoxicity was not specific to a macrophage cell line, we repeated the experiment in bone marrow derived macrophages prepared from BALB/c mice. As shown in **Figure 3**, Δ*lpcC*, Δ*manB*, and Δ*manC* were more toxic to primary macrophages than parent strain MFN245.

**Figure 4:**
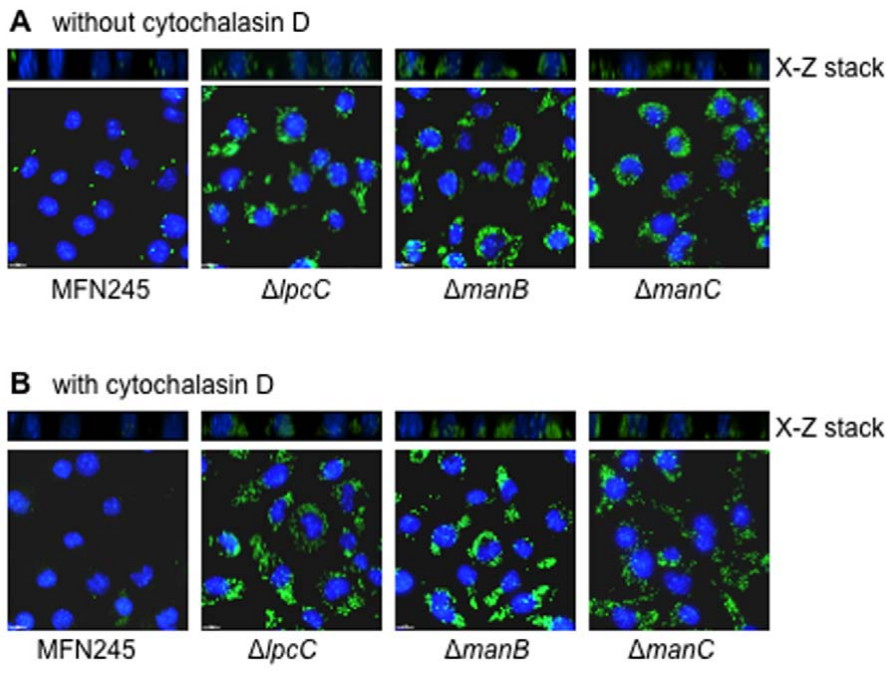
High numbers of mutant bacteria were visualized intracellularly in infected J774 macrophages even in the absence of actin polymerization. J774 macrophages were infected with the three deletion mutants or parental strain MFN245 in four-well microscope chambers for two hours at an MOI of 100 either in the absence (**A**) or presence (**B**) of cytD. The cells were fixed in 4% paraformaldehyde, permeabilized, and probed with a rabbit polyclonal antibody against *Francisella* followed by a secondary goat anti-rabbit antibody conjugated with Alexa 488 (green). J774 nuclei were identified by staining DNA with DAPI (blue). Cells were imaged with an Applied Precision DeltaVision deconvolution microscope system. Experiment was repeated six times with similar results and representative images are shown. Eukaryotic cell boundary can be observed in the phase-contrast images of the same fields. Scale bar 10 µm (lower left corner). X-Z stack images show that bacteria were within cells.

Because the hypercytotoxicity of the *lpcC*, *manB*, and *manC* mutant strains is not dependent upon actin polymerization, we wanted to determine if the bacteria were being internalized by the macrophages. Bacteria were visualized in macrophages infected with either one of the mutant strains or MFN245 using a fluorescently conjugated anti-*Francisella* antibody. To our surprise, we observed approximately one hundred times more Δ*lpcC*, Δ*manB*, and Δ*manC* bacteria in macrophages two hours after infection as compared to the parent strain (**Figure 4A**). In the presence of cytD, very few MFN245 bacteria were visualized internally. In contrast, a similar number of mutant bacteria were visualized inside macrophages in the presence of cytD as were visualized in the absence of cytD. (**Figure 4B**). Z sections prepared using the API Deltavision deconvolution microscope confirmed that the bacteria were inside the cell and not simply associated with the cell externally (**Figure 4**). Increasing the concentration of cytD to 50 µm did not inhibit the entry or cytotoxicity of the Δ*lpcC*, Δ*manB*, and Δ*manC* bacteria (data not shown). The localization of wild-type U112 bacteria in infected macrophages was indistinguishable from MFN245 under similar conditions (data not shown).

**Figure 5:**
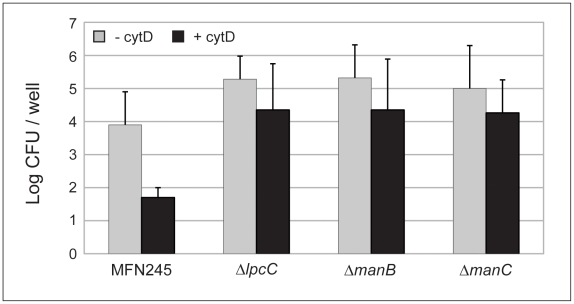
Inhibiting actin polymerization did not reduce the number of intracellular mutant bacteria. J774 macrophages were infected with Δ*lpcC*, Δ*manB* or Δ*manC* deletion mutants or parental strain MFN245 at a MOI of 100. Cells were infected either in the presence or absence of cytD. At two hours p.i., the macrophages were washed and treated with gentamicin to kill extracellular bacteria. Cells were lysed and the lysates plated on CHA plates. Colonies were counted two days after incubation and the numbers of CFU/well were calculated and converted to a log scale. Each column is an average of three individual infections (±s.d.). Repetition of this experiment yielded similar results.

As a way to quantitate the above results, we determined the number of intracellular bacteria in J774 macrophages after infection with Δ*lpcC*, Δ*manB*, Δ*manC* and MFN245 for two hours in the presence or absence of cytD. After two hours, the macrophages were washed and treated with gentamicin to remove extracellular bacteria and then lysed with saponin to determine the number of intracellular bacteria. As shown in **Figure 5**, the addition of cytD significantly lowered the uptake of the parent (p<0.01) but not the number of internal mutant bacteria (**Figure 5**). These data combined with the microscopy above suggest that some bacteria may be killed upon phagocytosis. Furthermore, at 8 hours p.i., the number of intracellular MFN245 bacteria was greater than the number of Δ*manB* and Δ*manC* (data not shown) suggesting that either the mutant strains grow more slowly than the wild-type strain or they are being killed inside macrophages.

### Increased cell uptake alone does not result in hypercytotoxicity

**Figure 6:**
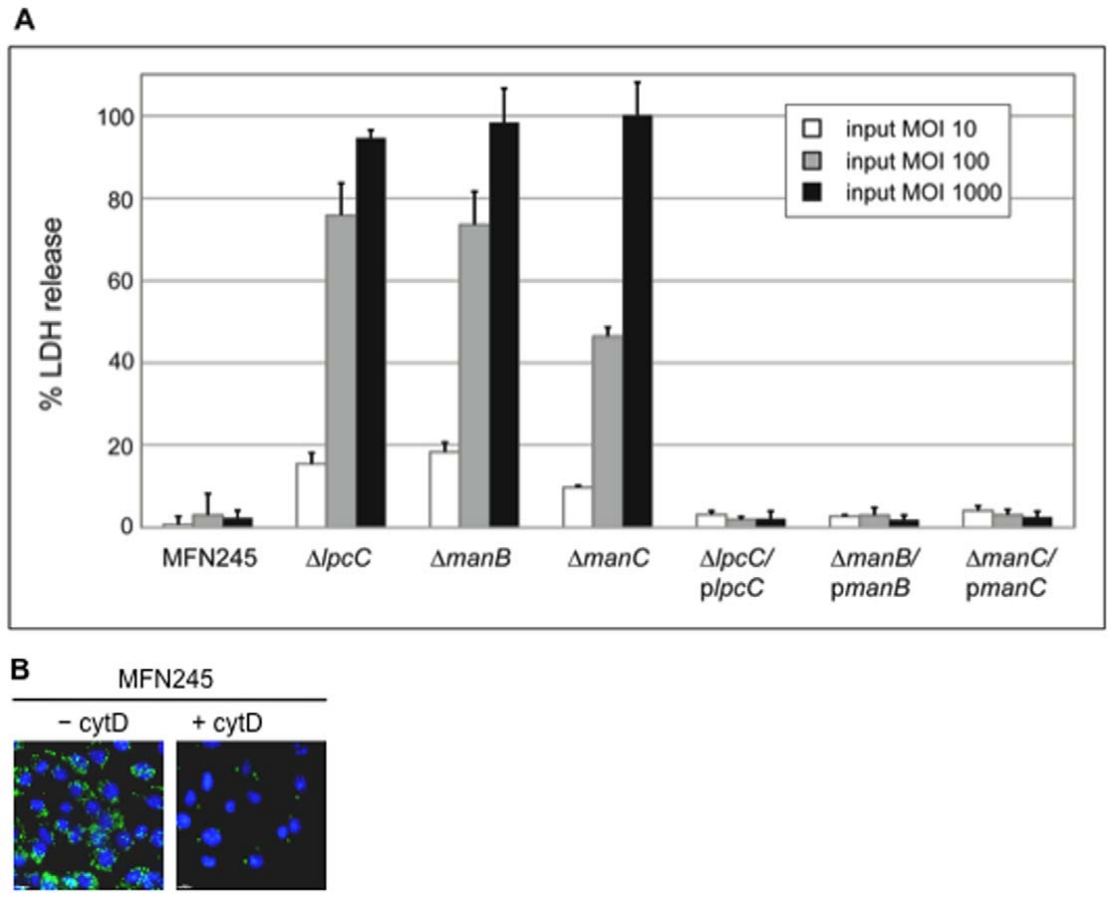
Increasing the number of internalized wild-type bacteria did not increase the cytotoxicity of the strain. (**A**) J774 macrophages were infected with Δ*lpcC*, Δ*manB*, Δ*manC*, or parental strain MFN245, as well as with complemented mutant strains expressing a wild-type copy of the gene *in trans*. The cells were infected for 10 hours at three different MOI. The level of LDH release from infected macrophages was determined as described in Figure 2. (**B**) J774 macrophages were infected with wild-type U112 at a MOI of 10,000 for two hours either in the presence or absence of cytD. *Francisella* (green) and macrophage nuclei (blue) were visualized as described in Figure 4. Both (A) and (B) were repeated a total of three times and yielded similar results.

We reasoned that the hypercytotoxic phenotype of Δ*lpcC*, Δ*manB*, and Δ*manC* might be solely due to the increased number of bacteria internalized by the macrophages. To test this hypothesis we varied the multiplicity of infection (MOI) for the wild-type parent and mutant strains and assayed for LDH release. Increasing the MOI of MFN245 did not increase the LDH released from infected J774 macrophages 10 hours p.i. (**Figure 6A**). LDH released from macrophages infected with the mutants increased with increasing MOI (**Figure 6A**) and was always higher than the LDH released following MFN245 infection at the same MOI. The increased LDH release in the mutants was abolished when the wild-type gene was expressed *in trans* indicating that the phenotype is specific to the deleted gene. The number of parent bacteria internalized at an input MOI of 10,000 was confirmed visually to be comparable to the mutant strains infected at a MOI of 100 (**Figure 6B** **vs.** **Figure 4**) and internalization of MFN245 at 10,000 MOI remained dependent upon actin polymerization (**Figure 6B**). These data indicate that the parental strain is remarkably non-toxic to cells even if there are very high numbers of intracellular bacteria.

### A bacterial surface structure is sufficient for increased macrophage invasions

**Figure 7:**
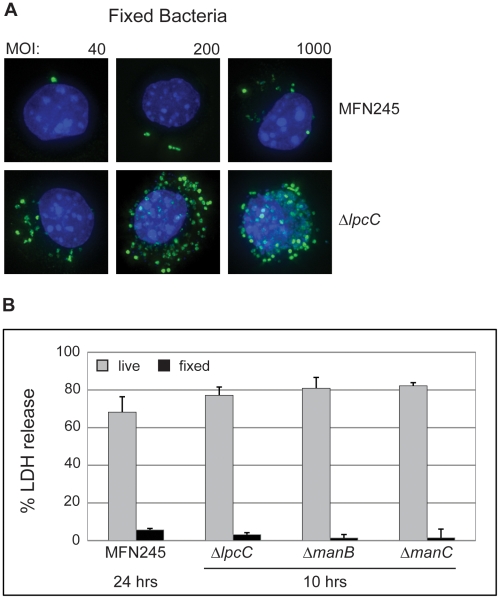
Dead bacteria do not promote cell death but are internalized similarly to live strains. (**A**) Formaldehyde-fixed Δ*lpcC* and MFN245 infected J774 macrophages at various MOI. *Francisella* (green) and macrophage nuclei (blue) were visualized as described in Figure 4. (**B**) J774 macrophages were infected with live mutant or parental bacteria and with strains that were fixed with 4% formaldehyde at a MOI of 100. LDH release was determined 12 hours p.i. for the mutant strains and 24 hours p.i. for wild-type strain as described in Figure 2. This experiment was repeated once with similar results.

In the case of *Salmonella* and many other intracellular pathogens, cell invasion requires synthesis of specific proteins. To determine if the exposed LPS core polysaccharide or some other exposed structure on the surface of the bacteria was sufficient to promote uptake, we determined whether bacteria treated with 4% formaldehyde, a cross-linking reagent, were internalized by J774 macrophages. Internalization of the mutant strains was compared to the parent strain MFN245 at the same input MOI by direct microscopic observations using polyclonal anti-*Francisella* antibodies to visualize the bacteria. As shown in **Figure 7A**, the number of internalized *lpcC* mutant bacteria is at least 10-fold higher than the parent at all concentrations of fixed bacteria. Similar results were observed for Δ*manB* and Δ*manC* strains (data not shown). These results indicate that the mutant bacteria possess a structure that promotes their uptake by macrophages, which is not exposed on the parent strain. Even though the dead parent and mutant strains were internalized by the macrophages, LDH release was low for all macrophages (**Figure 7B**). These results demonstrate that viable *Francisella* bacteria are necessary to promote macrophage cell death.

### Internalization of live *Francisella* bacteria is essential for macrophage killing

**Figure 8:**
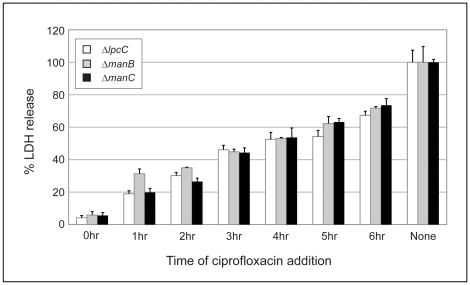
Viable bacteria are required for the cell toxicity observed in the mutant strains. J774 macrophages were infected with Δ*lpcC*, Δ*manB*, and Δ*manC* mutant strains at a MOI of 100. Ciprofloxacin, a bacteriocidal and host cell membrane permeable antibiotic, was added concurrent with infection (0 h) or at one of six time points following initial infection (1 h–6 h). LDH release levels were determined 12 hours p.i. as described in Figure 2 and compared to LDH release from infected macrophages not treated with ciprofloxacin (No). This experiment was repeated once with similar results.

Because fixed mutant strains failed to induce macrophage cell death, we wanted to determine how long the intracellular bacteria had to be viable for cell death to occur. Ciprofloxacin, a cell permeable bactericidal antibiotic, was added at different times following infection of J774 cells with Δ*lpcC*, Δ*manB*, or Δ*manC* strains. The macrophages were infected with the bacterial strains for two hours and then washed and treated with gentamicin to remove extracellular bacteria. Ciprofloxacin was added to infected macrophages at six separate time points after the initial treatment with gentamicin (hr 0) and then LDH release was measured 10 hours later (12 hours after initial infection). Without addition of ciprofloxacin, the Δ*lpcC*, Δ*manB*, and Δ*manC* bacteria killed >95% of J774 within 12 hours of infection at an input MOI of 100 (**Figure 8**). LDH released from cells infected with the parent at the same input MOI showed <10% LDH release at 12 hours p.i. (data not shown). The addition of ciprofloxacin simultaneously with gentamicin reduced LDH release by 90%. Ciprofloxacin added at one or two hours post-gentamicin treatment reduced LDH release by 80% or 70%, respectively (**Figure 8**). These results show that four to five hours after infection with Δ*lpcC*, Δ*manB*, or Δ*manC*, the majority of J774 macrophages are committed to cell death.

### *lpcC*, *manB*, and *manC* mutants have a shortened LPS structure

**Figure 9:**
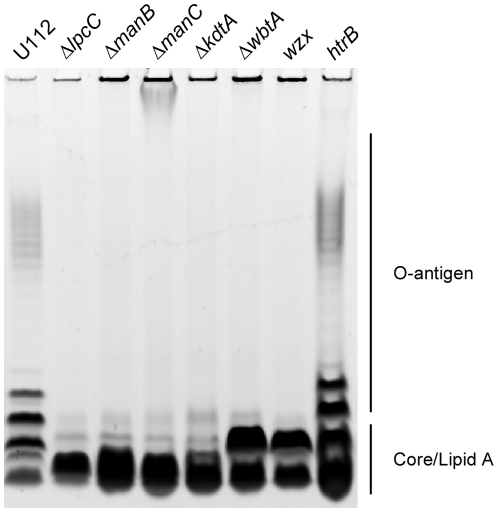
Lipopolysaccharides (LPS) prepared from Δ*lpcC*, Δ*manB*, Δ*manC*, and Δ*kdtA* lack the O-antigen and contain a defect in the core. Lipopolysaccharides were purified from U112; strains containing deletions in *lpcC* (FTN1253), *manB* (FTN1417), *manC* (FTN1418), *kdtA* (FTN1469), *wbtA* (FTN1431) and strains containing transposon mutations in *wzx* (FTN1420) and *htrB* (FTN0071) and analyzed on a gradient SDS-PAGE gel. The inverted image is shown in the figure.

Because *lpcC*, *manB*, and *manC* may be involved in LPS synthesis, we decided to analyze whether deleting these genes alters the LPS. The structure of the LPS can be partially deduced by its size following electrophoresis because it is assembled sequentially and as a consequence, mutants missing the core or O-antigens will migrate faster. The size of LPS synthesized in the deletion mutants was compared to wild-type strain U112, a strain that synthesizes a LPS without the core and O-antigen (Δ*kdtA*), two strains that synthesize a LPS lacking the O-antigen (Δ*wbtA*, *wzx*), and hypercytoxic transposon mutant *htrB*, which is predicted to alter the acylation of lipid A [26]. As shown in **Figure 9**, *lpcC*, *manB*, and *manC* mutant strains expressed a shortened LPS that resembled the LPS from the Δ*kdtA* mutant strain (**Figure 9**). These strains lack the O-antigen and likely contain a defect in the core. The O-antigen is also absent in Δ*wbtA* and *wzx* but these two strains seem to synthesize a complete core. The core and O-antigen were present in both wild-type U112 and transposon mutated *htrB*.

These data suggest that the LPS of the *lpcC*, *manB*, and *manC* deletion strains lack an O-antigen and have an absent or altered core. LPS is synthesized starting with lipid A, followed by the core, followed by the O-antigen; therefore, these three genes are involved in the synthesis of the core or of the O-antigens. As *lpcC*, *manB*, and *manC* are all involved in mannose biosynthesis, it is very likely that these enzymes are required for complete synthesis of the core (**Figure 1**). The exact changes in LPS structure after deleting *lpcC*, *manB*, and *manC* remain to be determined because altering LPS at one position may influence other modifications [24], [25].

### Other LPS biosynthesis mutants

**Figure 10:**
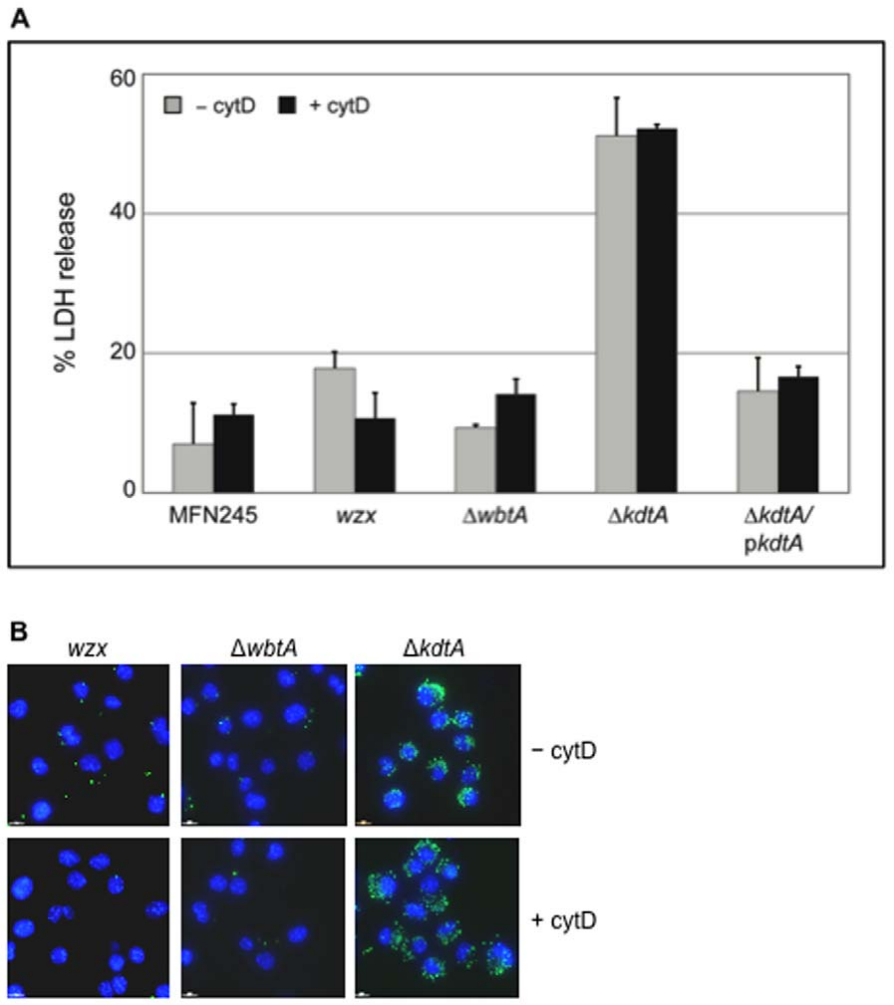
Deleting LPS biosynthesis gene *kdtA* resulted in a cytotoxicity and localization phenotype similar to the Δ*lpcC*, Δ*manB*, and Δ*manC* mutants. J774 macrophages were infected with Δ*wbtA* (FTN1431), Δ*kdtA* (FTN1469), transposon mutated FTN1420 (*wzx*), parental strain MFN245, or Δ*kdtA* complemented *in trans* with wild-type *kdtA* at a MOI of 100 for 10 hours either in the presence and absence of cytD. LDH release levels were determined as described in Figure 2. (**B**) *Francisella* (green) and macrophage nuclei (blue) were visualized in macrophages two hours after infection with the mutant strains as described in Figure 5. This experiment was repeated twice with similar results.

In our initial screening of the two-allele library, the other transposon mutants of LPS biosynthesis genes behaved like U112 or were hypocytotoxic, as judged by direct microscopic observation. However, as shown above, the LPS structure of Δ*lpcC*, Δ*manB*, and Δ*manC* is similar to Δ*kdtA* and both Δ*wbtA* and *wzx* synthesize a LPS lacking an O-antigen; therefore, we determined the cytotoxicity of these mutants by assaying LDH release from infected macrophages 10 hours after infection. As shown in **Figure 10A**, Δ*kdtA* killed infected macrophages as quickly as the three LPS mutants, Δ*lpcC*, Δ*manB*, and Δ*manC*. In contrast, Δ*wbtA* and *wzx* had a similar release of LDH as the parental strain MFN245. When a full-length copy of the *kdtA* gene was expressed *in trans* in Δ*kdtA*, LDH released from infected macrophages was not significantly different from the LDH released from MFN245-infected macrophages (**Figure 10A**). *KdtA* was probably not identified in the initial library screen because of polar effects on expression of downstream genes.

To determine whether internalization of Δ*wbtA*, Δ*kdtA*, and *wzx* was independent of actin polymerization, LDH release, and the number of intracellular bacteria were determined in infected J774 macrophages in the presence of cytD. High LDH release was observed for Δ*kdtA* at 10 hours, which was unchanged in the presence of cytD (**Figure 10A**). As shown by microscopy in **Figure 10B**, the number of intracellular mutant *wzx* and *wbtA* bacteria was similar to what was observed for MFN245 (**Figure 5A**) and reduced by the presence of cytD. In contrast, the number of intracellular Δ*kdtA* bacteria was greater than *wzx* and Δ*wbtA* and comparable to what was observed for Δ*lpcC*, Δ*manB*, and Δ*manC* (**Figure 4** **&** **Figure 10B**). Furthermore, the number of internal Δ*kdtA* bacteria was not affected by the presence of cytD (**Figure 10B**). These results show that modifications to the O-antigen, as observed with *wzx* and Δ*wbt*, are not sufficient to result in a hypercytotoxic phenotype and that alterations to the core were required.

## Discussion

Our results have identified four *F. novicida* genes involved in LPS core synthesis/assembly that have two surprising phenotypes when mutated. First, they are phagocytosed by macrophages at a much higher rate than the parent via a novel mechanism that does not require actin polymerization. Second, they are more toxic to macrophages than the parent. Phagocytosis of wild-type *Francisella* strains by primary murine macrophages and macrophage cell lines has previously been shown to require actin polymerization [14]–[16]. Our results represent one of the first examples of bacterial mutants that can enter macrophages without actin polymerization. However, as we discuss in more detail below, there is indirect evidence in other Gram-negative bacteria that mutations that alter the LPS may have phenotypes similar to the four mutants we identified in this manuscript. The increased phagocytosis observed in the *Francisella* mutants occurs even for bacteria that have been fixed with formaldehyde, suggesting that a *Francisella* surface structure(s) mediates uptake. In addition, formaldehyde-fixed *Francisella* treated with trypsin are also phagocytosed at a higher rate, suggesting that the bacterial ligand is not proteinacious (X.H. Lai and F. Heffron, unpub. data). While dead bacteria continue to be internalized at a high rate, the increased toxicity requires viable *Francisella*. Infected macrophages are not killed when they are treated with a bactericidal antibiotic immediately following phagocytosis of the mutant bacteria. In contrast, approximately half of the infected macrophages die if mutant bacteria remain alive four hours after infection. We assume infection with the mutant activates a macrophage cell death pathway, although which pathway is not yet known.

Several LPS mutants in addition to *lpcC*, *manB*, *manC* and *kdtA* were identified and studied in this work. A transposon insertion in *htrB*, which is 99% identical to *lpxL* and as such, is a late acylase that modifies lipid A [26], was found to be hypercytotoxic *in vitro* and attenuated in mice (**Table 1**). We found that this mutant was phagocytosed at a rate similar to that of the parent and synthesizes a LPS containing an O-antigen. Mutations in two other genes that are required for the addition of the O-antigen to the LPS including *wzx* and Δ*wbtA* were attenuated in mice [9,X.H. Lai and F. Heffron unpub data] but were not more cytotoxic to macrophages nor were they internalized at a higher rate (**Figure 10B**). In fact, none of the hypercytotoxic transposon mutants we identified through our screen were in genes required for O-antigen synthesis. The four mutants we identified as being more cytotoxic to macrophages and phagocytosed via an actin-independent pathway are all normally involved in synthesis of the LPS core. We speculate that the LPS structure remaining in these mutants alters the mechanism of infection; however, it is also possible that the shortened LPS exposes other surface structures that signal import. The uptake by macrophages may be mediated by a lectin receptor. One such lectin is dectin-1, which is present on macrophages and mediates resistance to fungal infections [27]. It binds 1,3 linked glucose oligomers, but it requires an 11–13mer oligosaccharide. A single 1,6 linked glucose is observed on the surface of lipid A in *F. novicida* but not other *Francisella* species.

### LPS mutants in other bacteria are phagocytosed at a high rate

Although this is the first example of a LPS mutant in *Francisella* being internalized at a higher rate, enhanced host cell uptake of LPS mutants in other microbes has been shown. Wick *et al*. found that peritoneal macrophages phagocytose rough mutants of *Salmonella typhimurium* four to ten times better than the smooth wild-type strain [28]. A recent paper described increased invasion of and adhesion to HeLa and MDCK cells for *Salmonella* mutants entirely lacking O-antigen [29]. Rough mutants of different *Brucella* species, including those due to mutation of the *manB* ortholog, are about 10 times more invasive to Vero cells [30], monocytes [31] and J774 macrophages [32], [33]. In the cases described above, we do not know whether the increased uptake was associated with increased cytotoxicity or whether the internalization was dependent upon actin polymerization. There are examples of microbes entering host cells in the absence of actin polymerization. Oelschlaeger *et al*. described entry into T24 bladder cells by some strains of *Campylobacter jejuni* and *Citrobacter freundii* that is independent of actin polymerization but requires microtubule assembly [34]. Schramm and Wyrick reported that disruption of microfilaments with cytochalasin D markedly reduces infection of host epithelial (HEC-1B) and fibroblast (McCoy) cells by *Chlamydia trachomatis* serovar E, but not serovar L2 [35]. Furthermore, in the presence of cytochalasin D, *Actinobacillus actinomycetemcomitans* strains SUNY 523 and 4065 exhibit enhanced ability to enter epithelial cells [36].

### The *Francisella* hypercytotoxic mutants are heterogeneous

We identified 28 *F. novicida* transposon mutants that increased the rate of cell death of infected macrophages. Even though all the mutants were hypercytotoxic, they differed in their attenuation in mice, their LPS patterns, and the rate and mechanism of their uptake in macrophages. Given the differences in phenotype, the exact mechanism of increased cell killing likely differs between the mutant strains. Weiss *et al*. identified two genes in *F. novicida* strain U112, FTN0757 (FTT0584) and FTN0720 (FTT0748), that are hypercytotoxic when mutated not because of a change in bacterial replication but rather because the mutants are more proinflammatory than the parental strain [37]. Similarly, a very recent report described a mutation in *tolC* of *F. tularensis* subsp. *holarctica* that is hypercytotoxic to macrophages and enhances a proinflammatory response from infected host cells [38]. In contrast, mutations in both FTN1592 (FTT0123, *oppB* oligopeptide permease) and FTN1186 (FTT1209c, *pepO*, endothelian converting enzyme) of *F. novicida* have been found to induce higher levels of cytotoxicity in macrophages because these mutants replicate to higher levels in macrophages than wild-type *Francisella* [18]. Among the mutants listed above only FTN0757 was identified in our screen because of its exceptionally strong hypercytotoxic phenotype. The cytotoxicity was not as robust in strains containing transposon mutations in FTN0720, FTN1209, FTN1592 and FTN1703; therefore, these strains were not pursued further.

The *Francisella lpcC*, *manB*, *manC* and *kdtA* mutants described in this study require viable bacteria for cell toxicity. Although these mutants were internalized at a high rate, this increase in initial internalization is not sufficient to explain the hypercytotoxic phenotype. The mutant strains are more cytotoxic as compared to the parental strain at all MOIs used for infection, and we did not observe an increase in macrophage cell death even when the number of intracellular parental bacteria was equivalent to the mutant strains. In addition, once internalized the number of mutant bacteria did not significantly increase eight hours p.i. (see Supporting Figure S1), which indicates that either the mutant bacteria replicate more slowly than the parental strain or are killed by the host cell at a rate that is approximately equal to their rate of replication. Therefore, the cytotoxicity of these strains is not the result of an increase in bacterial uptake or by an accelerated rate of replication. Based on previous studies, a possible explanation is that these mutants increase the inflammatory response by signaling through TLR4. However, primary macrophages prepared from TLR4^−^ mice (C3H/HeJ mice) were killed at about the same or greater rate than macrophages from congenic TLR^+^ mice (X.H. Lai and F. Heffron, unpubl. obs.). Other possible explanations that are being explored include measuring expression of different virulence factors after infection, comparing the phagocytic pathway of the mutants to the parent, and directly measuring the inflammatory pathways that are being stimulated.

The largest class of mutants identified in this study was that which affects type IV pili synthesis. Eleven of the thirteen *F. novicida pil* genes were found to be more cytotoxic to J774 cells than the wild-type U112 strain (**Table 1**). The finding that some of the *pil* genes are involved in both pilus production and protein secretion may help explain these results. U112 and LVS have been shown to secret proteins into culture supernatant via the type IV pilus [39], [40]. Among the *pil* genes, it has been shown that four mutants (*pilA, pilF*, *pilG*, and *pilQ*) reduced their protein secretion; mutations in *pilF* and *pilQ* resulted in fewer pili [39], [40]; and *pilA* and *pilF* mutants were less virulent in mice [39], [40]. It is possible that type IV pili are normally anti-inflammatory by themselves or alternatively, that *Francisella* secretes a factor via these structures that inhibits cell death. These possibilities are being tested by directly adding purified pilus system proteins to cells to determine if we can complement the cytotoxicity of a *pil* mutant, as well as by performing studies to determine if other secreted proteins could be anti-inflammatory.

The current study reports the finding of a class of hypercytotoxic mutants in *F. novicida* that are heterogeneous in their entry into macrophages, LPS structures and virulence *in vivo*. More extensive analyses of four of these mutants (*lpcC, manB*, *manC* and *kdtA*) showed that they were in genes necessary for synthesis of the LPS core. These *F. novicida* mutants utilize a highly efficient mechanism for entry into macrophages that does not involve eukaryotic microfilaments. The results suggest that *Francisella* has an alternative mechanism to enter macrophages, e.g. a vestigal mechanism left from the evolution of Gram-negative bacteria. The signal transduction pathways stimulated by the mutants vis-à-vis the parent, as well as the cognate cytoskeletal changes that take place, remain to be investigated. It is interesting that infection of lung alveolar type II (ATII) epithelial cells by *Francisella* is highly efficient and likely accounts for much of the pulmonary pathology [41], [42]. These same authors observed that the invasion of a lung ATII type epithelial cell line (TC-1) took place even with formaldehyde fixed *F. tularensis* LVS bacteria [43]. During LPS synthesis, some incomplete molecules are always expressed (R. Ernst, pers. comm.), perhaps resulting in bacteria that are taken up by ATII cells by the same mechanism that we describe.

## Materials and Methods

### Bacterial strains and growth conditions

*Francisella tularensis* ssp. *novicida* type strain U112, the *F. novicida* transposon two-allele mutant library [2], the restriction-deficient strain MFN245 [44], and the deletion mutant strains developed in this paper are stored at −80°C in tryptic soy broth (Becton, Dickinson and Company, Sparks, MD) plus 0.1% cysteine (TSBC) plus 10% DMSO. *Francisella* strains were cultured at 37°C in TSBC or on cysteine heart agar (CHA, Difco/Becton, Dickinson and Company) plates unless indicated below. Antibiotics used to select for *Francisella* transformants were kanamycin (20 µg/ml) and tetracycline (8 µg/ml). *E. coli* strains used to generate the allelic replacement and complementation plasmids were One Shot TOP10 Chemically Competent *E*. *coli* (Invitrogen, Carlsbad, CA) and TransforMax EC100D pir-116 Electrocompetent *E*. *coli* (Epicentre Biotechnologies, Madison, WI). *E*. *coli* transformants were grown in Luria-Bertani (LB) broth or agar containing kanamycin (60 µg/ml) or tetracycline (30 µg/ml).

### Culture and infection of J774 murine macrophage-like cells and murine bone marrow-derived macrophages (BMDM)

The J774 murine macrophage-like cells (American Type Culture Collection, Manassas, VA) were cultured in Ham's F10 medium (Gibco-BRL, Rockville, MD) supplemented with 10% fetal bovine serum (FBS) (Gibco-BRL), 1 mM nonessential amino acids (Gibco-BRL), and 0.2 mM sodium pyruvate (Gibco-BRL) at 37°C in the presence of 5% CO_2_. For infection, bacteria were added to 70% confluent cells in 6-, 24-, or 96-well culture dishes (Corning, Corning, NY) at the multiplicities of infection (MOI) indicated in the results section, and incubated at 37°C in the presence of 5% CO_2_. Two hours after infection, the cells were washed twice with phosphate-buffered saline (PBS), and cultured in Ham's F10 medium containing 10 µg/ml of gentamicin to prevent the growth of extracellular bacteria. For some experiments J774 cells were treated with 1 µg/ml cytochalasin D (Sigma, St. Louis, MO) 30 min prior to infection to inhibit actin filament polymerization.

BMDM were collected by flushing the femurs of BALB/c (TLR4+) mice with Dulbecco's modified Eagle's medium (DMEM, Invitrogen) and cultured in DMEM with 10% heat-inactivated FBS, 30% sterile filtered L-cell conditioned media (made in house), and penicillin/streptomycin (10,000 U/ml each) for six to seven days. The cells were split and infected as above for J774 macrophages.

### Lactate Dehydrogenase (LDH) release assay for cytopathogenicity

The LDH release assay was conducted as described [20]. Briefly, J774 macrophages (about 4-5×10^4^/well) seeded in 96-well culture plates were infected in triplicate with the transposon mutants, the non-polar deletion mutants, the restriction-deficient strain MFN245, or wild-type *F. novicida* U112 at the designated input MOI and washed at two hours post-infection (p.i.). At 12 and 24 hours p.i., the supernatants were removed and assayed for release of LDH using the CytoTox 96 nonradioactive cytotoxicity assay (Promega, Madison, WI). Cytotoxicity was determined by calculating LDH release as a percentage of the maximal amount released from macrophages lysed with detergent.

### Phase-contrast and fluorescent microscopy

J774 cells were infected as previously described [20] at the indicated input MOI, in four-well chamber plates (Nalgene Nunc/Thermo Scientific, Rochester, NY). After two hours, the cells were washed twice with PBS, fixed for one hour with 4% paraformaldehyde at 4°C. After three washes for 10 min in phosphate-buffered saline (PBS), the cells were permeabilized with 0.5% Triton X-100 (Sigma) in PBS for 20 min at room temperature, blocked with 5% FBS in PBS for 30 min, and incubated for one hour at 4°C with a polyclonal antibody against *F. tularensis* at a 1∶2,000 dilution (Becton, Dickinson and Company). After three washes for 10 min in PBS, the cells were again blocked with 5% FBS. A goat anti-rabbit antibody conjugated to Alexa 488 (Molecular Probes, Eugene, OR) was applied to the cells at a 1∶500 dilution for one hour at 4°C. The cells were again washed three times for 10 min in PBS and incubated with a 1∶1,000 dilution of FM 4-64 membrane stain (Molecular Probes) and 1∶1,000 dilution of DAPI DNA stain in PBS (Alexis Biochemicals, San Diego, CA) for 10 min at room temperature. The cells were washed twice with PBS and mounted in Fluormount-G antifade solution (Southern Biotechnology, Birmingham, AL), and images were obtained with an Applied Precision DeltaVision deconvolution microscope system (Advanced Precision Instruments, Issaquah, WA). All images were taken with a 60× objective. Stacks of 10 z-plane images that were 1 µm apart were captured at 1024×1024 pixels and deconvolved for seven iterations. Selected images were saved in TIFF format and imported into Adobe Photoshop to be formatted.

### Mouse studies

Six- to eight-week old female BALB/c mice were purchased from the Jackson Laboratory (Bar Harbor, ME) and acclimatized for one week. The animals were fed autoclaved food and water *ad libitum*. All experiments were performed in accordance with Animal Care and Use Committee guidelines (Institutional Approval B11220). Mutant strains were cultured in TSBC, OD normalized, and diluted with PBS. Mice were inoculated intraperitoneally (i.p.) with bacteria in 150 µl (total volume) of PBS and checked for signs of illness or death daily following infection for a total of 28 days. The 50% lethal doses (LD_50_) were calculated by the method of Reed and Muench [45].

### *in vitro* growth index assay

Overnight cultures of the transposon mutants and wild-type U112 strains were diluted in fresh TSBC to a starting OD_600_ of 0.05. At 18 hours post inoculation OD_600_ was taken and an *in vitro* growth index was calculated as (mutant OD_18h_ − mutant OD_0h_)/(wt OD_18h_ − wt OD_0h_) to determine if the mutant shows an obvious difference *in vitro* growth as compared to wild-type [21]. A value of ±30% (i.e., ±0.3) for the mutant strain is considered to be significantly different than the wild-type.

### Construction and complementation of deletions in *F. novicida*

All deletions were generated in *F. novicida* mutant strain MFN245, a quadruple mutant that substantially reduces the restriction barrier and thereby increases the efficiency of transformation [44]. The cytotoxicity and virulence of this modified host strain in mice is comparable to U112 (X.-H. Lai and F. Heffron, unpub. data). Plasmid pKD13 [46] was modified such that expression of kanamycin was more efficient in *Francisella* and segments of *Francisella* DNA would be easier to clone into the vector. First, the *groE* promoter was amplified from *F. novicida* genomic DNA by PCR using oligonucleotides 5′ -CGCGGATCCGTATGGATTAGTCGAGC and 5′- CGCGGATCCTGCACGACGAACTAATACTC. The oligonucleotides also contained a recognition site for *Bam*HI at the 5′ end. The DNA fragment containing the *groE* promoter was digested with *Bam*HI and ligated into the *Bgl*II site directly upstream of the *kan* gene in pKD13. A pKD13 plasmid with the *groE* promoter in the correct orientation and free of PCR errors was identified by sequence analysis. Second, complementary pairs of oligonucleotides were annealed to generate DNA fragments containing *Apa*I and *Sbf*I restriction sites (5′-TCGAGGGCCCGCACCTGCAGGGC and 5′-TCGACCTGCAGGTGCGGGCCC) and *Asc*I and *Sma*I restriction sites (5′-CTAGAGGCGCGCCGCCCCGGG and 5′-CTAGCCCGGGGCGGCGCGCCT). After annealing, the complementary oligonucleotides had single-strand 5′ overhangs that allowed the fragments to be cloned into pKD13 digested with *Sal*I and *Avr*II, respectively. In the final modified pKD13 plasmid, both the *Apa*I and *Sma*I restriction sites were most distal to the FRT sites. For each gene to be deleted, two sets of oligonucleotide pairs were designed (**Table 3**). The first pair (labeled Up F and Up R) amplifies the first 50–70 bp of the open reading frame (ORF) plus ∼500 bp upstream and includes recognition sites for *Apa*I and *Sbf*I. The second pair (labeled Down F and Down R) amplifies the last 50–70 bp of the ORF plus ∼500 bp downstream and includes recognition sites for *Asc*I and *Sma*I.

**Table 3:** Oligonucleotides used in this study.

Primer	Sequence
FTN1253 up F	5′-GATCGGGCCCCCATCGTATAGCTTGCCAAT-3′
FTN1253 up R	5′-GATCCCTGCAGGTACCAGAAAATCTACGTCCTAGTGAT-3′
FTN1253 down F	5′-GATCGGCGCGCCTGAAGCTGAAGGGATTCAACAA-3′
FTN1253 down R	5′-GATCCCCGGGTGCTCCTACTTATGATTGGCATC-3′
FTN1417 up F	5′-GATCGGGCCCTATTTACGCTCGCATGATCG-3′
FTN1417 up R	5′-GATCCCTGCAGGTACCAAACTTTACGCCGCTAGA-3′
FTN1417 down F	5′-GATCGGCGCGCCGGCTAGTGATGAGCAGGCAAA-3′
FTN1417 down R	5′-GATCCCCGGGCTTTGGGTGCTGCGTAAGAT-3′
FTN1418 up F	5′-GATCGGGCCCAAACCAGAAAATGCTCCACA-3′
FTN1418 up R	5′-GACTCCTGCAGGATAGTGGCCATAGCCTTGAGC-3′
FTN1418 down F	5′-GATCGGGCGCGCCGCAAGTGGGAGAATATATAAGTGA-3′
FTN1418 down R	5′-GATCCCCGGGTGCTTGCTTACTAGGCTCTGG-3′
*kdtA* up F	5′-GATCGGGCCCCCTCAAACTGATTTTAATGTTCCTGACGC-3′
*kdtA* up R	5′-GATCCCTGCAGGGCGAATCTCTCAGCCCATCTTTTTCTG-3′
*kdtA* down F	5′-GATCGGCGCGCCTTAAAAAGCCATAGTGATGTACTCGAAAAACAG-3′
*kdtA* down R	5′-GATCCCCGGGCCTCAATATCTAGTTGTTGACCACCAACC-3′
*wbtA* up F	5′-GATCGGGCCCAACACCTTAGCACTGGTGATGAAGAAGTAAC-3′
*wbtA* up R	5′-GATCCCTGCAGGACTATTATTACCACGAAATTAAGCGTTCTATTATCG-3′
*wbtA* down F	5′-GATCGGCGCGCCCAGCTTGTGATATTAAAGAAAATTGTTCCG-3′
*wbtA* down R	5′-GATCCCCGGGCTTGTAGAAACTACCTAAACTTTCAGCAGCATC-3′
FTN1253 complete F	5′-GATCGCGGCCGCTTTACCATCGTATAGCTTGCCAATAGTCG-3′
FTN1253 complete R	5′-GATCGGGCCCTGATAATAATGAAAATCTTGTCACTAAAGTCACCC-3′
FTN1417/18 complete F	5′-GATCGCGGCCGCATGAATATAAACCAGAAAATGCTCCACATTC-3′
FTN1417/18 complete R	5′-GATCGGGCCCCGAAAATGAAAGGCTCACTAACTAATGAAGAGTTC-3′
FTN1418 complete F	5′-GATCGCGGCCGCTGTAAACTAATGGATGAATATAAACCAGAAAATGC-3′
FTN1418 complete R	5′-GATCGGGCCCCGCCGAAACAAGACCTCTAACTCCACTG-3′
*kdtA* complete F	5′-GATCGCGGCCGCTCCTGACGCTGATGAAATTG-3′
*kdtA* complete R	5′-GATCGGGCCCGCCCGCTAAGATTGCAGTAG-3′

For one-step allelic replacement, pKD13 containing the *Francisella* fragments was digested with *Apa*I and *Sma*I and transformed into MFN245 as described previously [47]. Kanamycin resistant transformants were streaked for single colonies and correct integration of only the linear fragment was verified by PCR. Although the gene deletions marked with kanamycin are in-frame, we proceeded to remove the kanamycin resistance gene by transforming the temperature-sensitive plasmid pFFLP [44], which expresses the flippase recombination enzyme, into the kanamycin resistant colonies by electroporation as described in [48]. Briefly, a 10 ml culture in Mueller-Hinton (MH) broth containing 0.1% glucose, 0.025% ferric pyrophosphate, and 2% IsoVitaleX (Becton Dickinson) was inoculated to an OD of ∼0.15 using overnight cultures of each of the deletion mutants. After approximately four hours of growth the cultures (OD between 0.3 to 0.5) were washed twice with 10–15 ml of 0.5 M sucrose (Fisher Scientific) and suspended in 200 µl of 0.5 M sucrose. One microliter pFFLP (500 ng/µl) was mixed with 200 µl of cells, incubated at room temperature for 10 min, transferred to a 0.2-mm cuvette and electroporated using a GenePulser (BioRad) at 2.5 kV, 25 µF, and 600Ω. Following electroporation, cells were suspended in 1 ml of MH broth and incubated at 30°C for two hours before plating on CHA plates containing 8 µg/ml tetracycline. As detailed in Gallagher LA *et al*. (2008), tetracyline resistant transformants were streaked for single colonies on tetracycline and grown at 30°C. Single colonies were transferred to CHA, grown at 37°C and tested for kanamycin sensitivity. Kanamycin sensitive colonies were streaked again for single colonies on CHA, grown at 37°C and tested for tetracycline sensitivity. The tetracycline sensitive colonies, which indicate loss of the pFFLP plasmid, were used in the experimental analyses. The resolved deletion was confirmed by PCR and sequencing.

The gene deletions in Δ*lpcC*, Δ*manB*, Δ*manC* and Δ*kdtA* strains were complemented *in trans* by transforming into the mutant strains plasmids that express the wild-type gene. The promoter and ORF of *lpcC* (FTN1253), *manC* (FTN1418) and *kdtA* (FTN1469) were amplified by PCR from *F. novicida* genomic DNA using oligonucleotides complementary to ∼500 bp upstream of the start codon and 100–300 bp downstream of the stop codon. *manB* (FTN1417) lies downstream of *manC* in an operon; in order to express *manB* from its endogenous promoter, we designed oligonucleotides that amplified 503 bp upstream of *manC* and 403 bp downstream of *manB*. The resulting FTN1417/18 PCR fragment contains the entire operon. The oligonucleotides used to amplify the wild-type copies of *lpcC*, *manB*, *manC* and *kdtA* are described in **Table 3** and contain recognition sites for *Not*I and *Apa*I. DNA fragments digested with *Not*I and *Apa*I were cloned into similar sites into a modified pKK202 that contains unique restriction sites for *Not*I, *Xho*I and *Sfi*I [20].

### LPS gel analysis

The LPS from *F. novicida* wild-type and mutant strains was isolated from whole cells after growth in TSBC for 24 hours. Bacteria (1 ml) were pelleted, resuspended in 100 µl lysis buffer (187 mM Tris-HCl, pH 6.8, 2% SDS, 10% glycerol, 4% 2-mercaptoethanol, 0.03% bromophenol blue), heated to 100°C for 10 minutes, and cooled to room temperature. Subsequently, 25 µg proteinase K was added to each sample and incubated at 60°C for one hour. Finally, samples were incubated at 100°C for 10 minutes, cooled briefly on ice and subjected to sodium dodecyl sulfate (SDS)-polyacrylamide gel electrophoresis (PAGE) using Bio-Rad Ready Gel precast 10–20% gradient Tris-Tricine/Peptide polyacrylamide gels (Hercules, CA). After electrophoresis, the LPS was stained with Pro-Q Emerald 300 LPS stain kits (Invitrogen, Carlsbad, CA), according to the manufacturer's recommendation, visualized, and photographed using the AlphaImager digital imaging system (Alpha Innotech Corp., San Leandro, CA).

### Statistics

Statistical significance of data was determined by using an unpaired analysis of variance and the Tukey-Kramer multiple-comparisons test (GraphPad Prism 4, San Diego, CA).

## Supporting Information

Figure S1 Mutant bacteria are internalized at higher levels than parent bacteria but do not replicate robustly inside host cells. RAW264.7 macrophages were infected with parent strain MFN245 or the ΔlpcC mutant strain at an input MOI of 100. One hour after infection, the cells were washed three times with PBS and incubated in DMEM containing 100 µg/ml gentimicin. One hour later, the cells were washed again and incubated in DMEM containing 10 µg/ml gentimicin. At two, four, or eight hours p.i., the macrophages were lysed in TSBC with 0.5% saponin, and intracellular bacteria were quantified by plating. Each infection was performed in triplicate and with a mock-infected control. MFN245 was less abundant intracellularly than ΔlpcC at two hours (p<0.001), four hours (p<0.05), and eight hours (p<0.05) p.i. The number of internalized ΔlpcC at two hours was not statistically significantly different from the level of internalized ΔlpcC observed at either four or eight hours p.i.
(0.68 MB TIF)

## References

[1] Sjostedt A, Brenner DJ, Krieg NR, Staley JT, Garrity GM (2005). Family III. *Francisellaceae*.. Bergey's Manual of Systematic Bacteriology, 2nd ed, vol. 2 (*The Proteobacteria*), part B (*The Gammaproteobacteria*).

[2] Gallagher LA, Ramage E, Jacobs MA, Kaul R, Brittnacher M (2007). A comprehensive transposon mutant library of Francisella novicida, a bioweapon surrogate.. Proc Natl Acad Sci U S A.

[3] Raetz CR, Whitfield C (2002). Lipopolysaccharide endotoxins.. Annu Rev Biochem.

[4] Raetz CR, Guan Z, Ingram BO, Six DA, Song F (2009). Discovery of new biosynthetic pathways: the lipid A story.. J Lipid Res.

[5] Gunn JS, Ernst RK (2007). The structure and function of Francisella lipopolysaccharide.. Ann N Y Acad Sci.

[6] Prior JL, Prior RG, Hitchen PG, Diaper H, Griffin KF (2003). Characterization of the O antigen gene cluster and structural analysis of the O antigen of Francisella tularensis subsp. tularensis.. J Med Microbiol.

[7] Thomas RM, Titball RW, Oyston PC, Griffin K, Waters E (2007). The immunologically distinct O antigens from Francisella tularensis subspecies tularensis and Francisella novicida are both virulence determinants and protective antigens.. Infect Immun.

[8] Guo H, Yi W, Song JK, Wang PG (2008). Current understanding on biosynthesis of microbial polysaccharides.. Curr Top Med Chem.

[9] Raynaud C, Meibom KL, Lety MA, Dubail I, Candela T (2007). Role of the wbt locus of Francisella tularensis in lipopolysaccharide O-antigen biogenesis and pathogenicity.. Infect Immun.

[10] Wang X, Ribeiro AA, Guan Z, McGrath SC, Cotter RJ (2006). Structure and biosynthesis of free lipid A molecules that replace lipopolysaccharide in Francisella tularensis subsp. novicida.. Biochemistry.

[11] Hajjar AM, Harvey MD, Shaffer SA, Goodlett DR, Sjostedt A (2006). Lack of in vitro and in vivo recognition of Francisella tularensis subspecies lipopolysaccharide by Toll-like receptors.. Infect Immun.

[12] Chang CC, Nowotny (1975). Relation of structure to function in bacterial O-antigens–VII. Endotoxicity of ‘lipid A’.. Immunochemistry.

[13] Kanipes MI, Ribeiro AA, Lin S, Cotter RJ, Raetz CR (2003). A mannosyl transferase required for lipopolysaccharide inner core assembly in Rhizobium leguminosarum. Purification, substrate specificity, and expression in Salmonella waaC mutants.. J Biol Chem.

[14] Clemens DL, Lee BY, Horwitz MA (2005). Francisella tularensis enters macrophages via a novel process involving pseudopod loops.. Infect Immun.

[15] Lai XH, Golovliov I, Sjostedt A (2001). Francisella tularensis induces cytopathogenicity and apoptosis in murine macrophages via a mechanism that requires intracellular bacterial multiplication.. Infect Immun.

[16] Lindemann SR, McLendon MK, Apicella MA, Jones BD (2007). An in vitro model system used to study adherence and invasion of Francisella tularensis live vaccine strain in nonphagocytic cells.. Infect Immun.

[17] Anthony LD, Burke RD, Nano FE (1991). Growth of Francisella spp. in rodent macrophages.. Infect Immun.

[18] Brotcke A, Weiss DS, Kim CC, Chain P, Malfatti S (2006). Identification of MglA-regulated genes reveals novel virulence factors in Francisella tularensis.. Infect Immun.

[19] Weiss DS, Henry T, Monack DM (2007). Francisella tularensis: activation of the inflammasome.. Ann N Y Acad Sci.

[20] Tempel R, Lai XH, Crosa L, Kozlowicz B, Heffron F (2006). Attenuated Francisella novicida transposon mutants protect mice against wild-type challenge.. Infect Immun.

[21] Su J, Yang J, Zhao D, Kawula TH, Banas JA (2007). Genome-wide identification of Francisella tularensis virulence determinants.. Infect Immun.

[22] Hager AJ, Bolton DL, Pelletier MR, Brittnacher MJ, Gallagher LA (2006). Type IV pili-mediated secretion modulates Francisella virulence.. Mol Microbiol.

[23] Gavrilin MA, Bouakl IJ, Knatz NL, Duncan MD, Hall MW (2006). Internalization and phagosome escape required for Francisella to induce human monocyte IL-1beta processing and release.. Proc Natl Acad Sci U S A.

[24] Raetz CR, Garrett TA, Reynolds CM, Shaw WA, Moore JD (2006). Kdo2-Lipid A of Escherichia coli, a defined endotoxin that activates macrophages via TLR-4.. J Lipid Res.

[25] Stead C, Tran A, Ferguson DJ, McGrath S, Cotter R (2005). A novel 3-deoxy-D-manno-octulosonic acid (Kdo) hydrolase that removes the outer Kdo sugar of Helicobacter pylori lipopolysaccharide.. J Bacteriol.

[26] McLendon MK, Schilling B, Hunt JR, Apicella MA, Gibson BW (2007). Identification of LpxL, a late acyltransferase of Francisella tularensis.. Infect Immun.

[27] Torrelles JB, Azad AK, Schlesinger LS (2006). Fine discrimination in the recognition of individual species of phosphatidyl-myo-inositol mannosides from Mycobacterium tuberculosis by C-type lectin pattern recognition receptors.. J Immunol.

[28] Wick MJ, Harding CV, Normark SJ, Pfeifer JD (1994). Parameters that influence the efficiency of processing antigenic epitopes expressed in Salmonella typhimurium.. Infect Immun.

[29] Holzer SU, Schlumberger MC, Jackel D, Hensel M (2009). Effect of the O-antigen length of lipopolysaccharide on the functions of Type III secretion systems in Salmonella enterica.. Infect Immun.

[30] Detilleux PG, Deyoe BL, Cheville NF (1990). Penetration and intracellular growth of Brucella abortus in nonphagocytic cells in vitro.. Infect Immun.

[31] Monreal D, Grillo MJ, Gonzalez D, Marin CM, De Miguel MJ (2003). Characterization of Brucella abortus O-polysaccharide and core lipopolysaccharide mutants and demonstration that a complete core is required for rough vaccines to be efficient against Brucella abortus and Brucella ovis in the mouse model.. Infect Immun.

[32] Pei J, Ficht TA (2004). Brucella abortus rough mutants are cytopathic for macrophages in culture.. Infect Immun.

[33] Pei J, Wu Q, Kahl-McDonagh M, Ficht TA (2008). Cytotoxicity in macrophages infected with rough Brucella mutants is type IV secretion system dependent.. Infect Immun.

[34] Oelschlaeger TA, Guerry P, Kopecko DJ (1993). Unusual microtubule-dependent endocytosis mechanisms triggered by Campylobacter jejuni and Citrobacter freundii.. Proc Natl Acad Sci U S A.

[35] Schramm N, Wyrick PB (1995). Cytoskeletal requirements in Chlamydia trachomatis infection of host cells.. Infect Immun.

[36] Brissette CA, Fives-Taylor PM (1999). Actinobacillus actinomycetemcomitans may utilize either actin-dependent or actin-independent mechanisms of invasion.. Oral Microbiol Immunol.

[37] Weiss DS, Brotcke A, Henry T, Margolis JJ, Chan K (2007). In vivo negative selection screen identifies genes required for Francisella virulence.. Proc Natl Acad Sci U S A.

[38] Platz GJ, Bublitz DC, Mena P, Benach JL, Furie MB (2010). A tolC mutant of Francisella tularensis is hypercytotoxic compared to the wild type and elicits increased proinflammatory responses from host cells.. Infect Immun.

[39] Chakraborty S, Monfett M, Maier TM, Benach JL, Frank DW (2008). Type IV pili in Francisella tularensis: roles of pilF and pilT in fiber assembly, host cell adherence, and virulence.. Infect Immun.

[40] Zogaj X, Chakraborty S, Liu J, Thanassi DG, Klose KE (2008). Characterization of the Francisella tularensis subsp. novicida type IV pilus.. Microbiology.

[41] Hall JD, Craven RR, Fuller JR, Pickles RJ, Kawula TH (2007). Francisella tularensis replicates within alveolar type II epithelial cells in vitro and in vivo following inhalation.. Infect Immun.

[42] Hall JD, Woolard MD, Gunn BM, Craven RR, Taft-Benz S (2008). Infected-host-cell repertoire and cellular response in the lung following inhalation of Francisella tularensis Schu S4, LVS, or U112.. Infect Immun.

[43] Craven RR, Hall JD, Fuller JR, Taft-Benz S, Kawula TH (2008). Francisella tularensis invasion of lung epithelial cells.. Infect Immun.

[44] Gallagher LA, McKevitt M, Ramage ER, Manoil C (2008). Genetic dissection of the Francisella novicida restriction barrier.. J Bacteriol.

[45] Reed LJ, Muench H (1935). A sinple method of estimating fifty percent endpoints.. Am J Hyg.

[46] Datsenko KA, Wanner BL (2000). One-step inactivation of chromosomal genes in Escherichia coli K-12 using PCR products.. Proc Natl Acad Sci U S A.

[47] Ludu JS, Nix EB, Duplantis BN, de Bruin OM, Gallagher LA (2008). Genetic elements for selection, deletion mutagenesis and complementation in Francisella spp.. FEMS Microbiol Lett.

[48] Maier TM, Havig A, Casey M, Nano FE, Frank DW (2004). Construction and characterization of a highly efficient Francisella shuttle plasmid.. Appl Environ Microbiol.

