# Supplementary figures and images for: Mutations of Francisella novicida that Alter the Mechanism of Its Phagocytosis by Murine Macrophages
Source: PLoS One. 2010 Jul 29;5(7):e11857. doi: 10.1371/journal.pone.0011857 (PMC2912274; doi:10.1371/journal.pone.0011857)

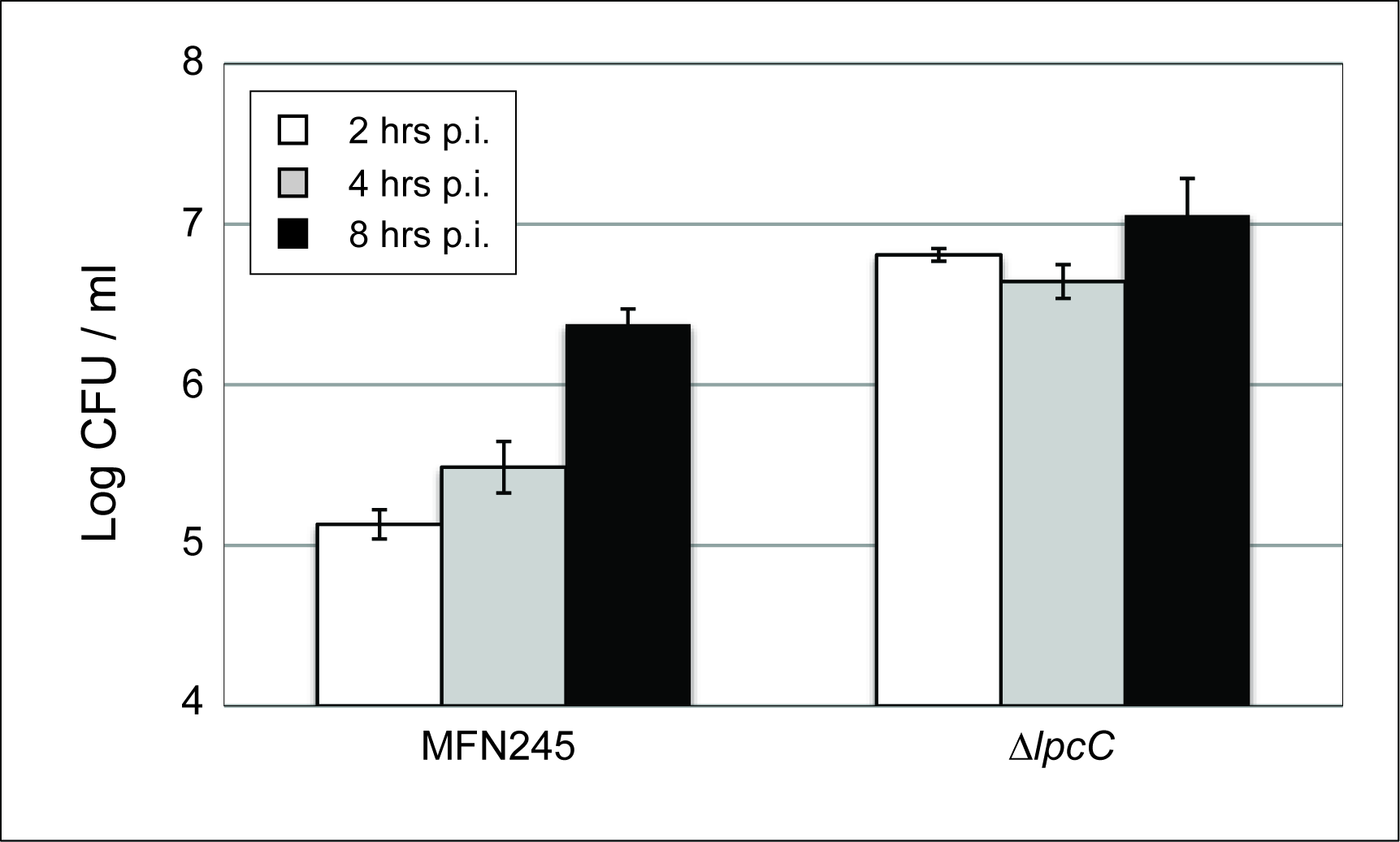

Supplement: Figure S1 — Mutant bacteria are internalized at higher levels than parent bacteria but do not replicate robustly inside host cells. RAW264.7 macrophages were infected with parent strain MFN245 or the ΔlpcC mutant strain at an input MOI of 100. One hour after infection, the cells were washed three times with PBS and incubated in DMEM containing 100 µg/ml gentimicin. One hour later, the cells were washed again and incubated in DMEM containing 10 µg/ml gentimicin. At two, four, or eight hours p.i., the macrophages were lysed in TSBC with 0.5% saponin, and intracellular bacteria were quantified by plating. Each infection was performed in triplicate and with a mock-infected control. MFN245 was less abundant intracellularly than ΔlpcC at two hours (p<0.001), four hours (p<0.05), and eight hours (p<0.05) p.i. The number of internalized ΔlpcC at two hours was not statistically significantly different from the level of internalized ΔlpcC observed at either four or eight hours p.i. (0.68 MB TIF) [file pone.0011857.s001.tif]
